# Incorporation of Resveratrol in Polymeric Nanogel for Improvement of Its Protective Effects on Cellular and Microsomal Oxidative Stress Models

**DOI:** 10.3390/gels9060450

**Published:** 2023-05-30

**Authors:** Lyubomira Radeva, Denitsa Stefanova, Yordan Yordanov, Katya Kamenova, Petar D. Petrov, Maya K. Marinova, Svilen P. Simeonov, Magdalena Kondeva-Burdina, Virginia Tzankova, Krassimira Yoncheva

**Affiliations:** 1 Faculty of Pharmacy, Medical University of Sofia, 1000 Sofia, Bulgaria; 2 Institute of Polymers, Bulgarian Academy of Sciences, 1113 Sofia, Bulgaria; 3 Institute of Organic Chemistry with Centre of Phytochemistry, Bulgarian Academy of Sciences, 1113 Sofia, Bulgaria; 4 Research Institute for Medicines (iMed.ULisboa), Faculty of Pharmacy, Universidade de Lisboa, Av. Prof. Gama Pinto, 1649-003 Lisbon, Portugal

**Keywords:** natural nanogel, resveratrol, protective effects, fibroblasts, neuroblastoma cells, lipid peroxidation

## Abstract

Nanogels are attractive drug delivery systems that provide high loading capacity for drug molecules, improve their stability, and increase cellular uptake. Natural antioxidants, especially polyphenols such as resveratrol, are distinguished by low aqueous solubility, which hinders therapeutic activity. Thus, in the present study, resveratrol was incorporated into nanogel particles, aiming to improve its protective effects in vitro. The nanogel was prepared from natural substances via esterification of citric acid and pentane-1,2,5-triol. High encapsulation efficiency (94.5%) was achieved by applying the solvent evaporation method. Dynamic light scattering, atomic force microscopy, and transmission electron microscopy revealed that the resveratrol-loaded nanogel particles were spherical in shape with nanoscopic dimensions (220 nm). In vitro release tests showed that a complete release of resveratrol was achieved for 24 h, whereas at the same time the non-encapsulated drug was poorly dissolved. The protective effect of the encapsulated resveratrol against oxidative stress in fibroblast and neuroblastoma cells was significantly stronger compared to the non-encapsulated drug. Similarly, the protection in a model of iron/ascorbic acid-induced lipid peroxidation on rat liver and brain microsomes was higher with the encapsulated resveratrol. In conclusion, embedding resveratrol in this newly developed nanogel improved its biopharmaceutical properties and protective effects in oxidative stress models.

## 1. Introduction

Nanoparticles are intensively investigated drug delivery systems that can improve the biopharmaceutical and pharmacological properties of different substances. They can protect the loaded drugs from unfavorable in vitro and in vivo conditions. It is well known that nanoparticles could increase the amount of drug that reaches the targeted tissues [1,2]. The small diameter of nanoparticles, particularly under 200 nm, mediates the enhanced permeability and retention (EPR) effect [3]. Nanogels are a type of nanoscale drug delivery system that combines the advantages of nanoparticles and hydrogels. They are defined as three-dimensional networks that can be obtained by hydrophilic polymers via simultaneous or sequential polymerization and cross-linking. Nanogels are considered to be non-toxic, biocompatible, and biodegradable. Furthermore, the nanogels could provide a high loading of active substances because of the different types of their structures, e.g., hollow, core/shell, and layer-by-layer structures. For instance, the encapsulation efficiency of doxorubicin in carboxymethyl chitosan nanogel approximated 95% [4]. The hydrophobic drug camptothecin was included in a core-shell nanogel prepared from poly(D,L-lactic acid)/poly(ethylene glycol)/poly(D,L-lactic acid) triblock copolymer, reaching an encapsulation efficiency of 80% [5]. Xing et al. reported high loading of isoniazid (668 mg/g) in hollow nanogels based on poly(acrylic acid) and poly(N-isopropylacrylamide) [6]. Depending on the type of polymers, the nanogels could be responsive to external stimuli such as pH, ionic strength, temperature, etc. For example, doxorubicin was loaded into a lactoferrin/phenylboronic acid-functionalized hyaluronic acid nanogel that was cross-linked via obtaining disulfide bonds [7]. The study achieved a nanosystem that was sensitive to reduction, and in particular, it ensured rapid drug release at high concentrations of glutathione. The cytotoxicity of the drug, cellular uptake, and brain permeability were enhanced in vitro in glioblastoma (G422) cells and in vivo in rats and mice. The antioxidant edaravone was encapsulated in glutathione-conjugated poly(methacrylic acid) nanogel, aiming to overcome its low aqueous solubility, stability, and bioavailability. In vivo tests on Wistar rats showed enhanced poststroke antioxidant and neuroprotective effects, better penetration through the blood–brain barrier and non-toxicity [8]. A thermosensitive poly(N-isopropylacrylamine-co-acrylic acid) nanogel was developed for the delivery of human cardiac stem cells. The study group observed the absence of systemic inflammation or local T-cell infiltrations in immunocompetent mice, preservation of cardiac function, and reduction of scar sizes in mouse and pig models of myocardial infarction [9]. Rodrigues da Silva et al. prepared nanoparticles from lipids and sodium alginate, which form nanogels in situ, and loaded them with bupivacaine. They improved the stability of the drug, increased the concentration that reached the site of action, and prolonged the release [10].

Nowadays, natural antioxidants are widely investigated for their ability to influence diseases related to high oxidative stress levels. Resveratrol is a polyphenol that is produced by a non-specific response to injury or microbial attack in members of the *Vitaceae* family [11]. It possesses various biological activities, in particular antioxidant, cardioprotective, vasorelaxant, anti-inflammatory, neuroprotective effects, and others [12,13,14,15]. The antioxidant activity is related to resveratrol’s ability to scavenge radicals such as nitric oxide, nitrogen dioxide, hydrogen peroxide, superoxide, and hydroxyl radicals. In addition, resveratrol could induce the expression of antioxidant enzymes —catalase, glutathione-S-transferase, glutathione peroxidase, superoxide dismutase, and NADPH quinine oxidoreductase [16]. It has been reported that the para-hydroxyl group in the structure of the polyphenol is most responsible for its scavenging activity [17]. In human erythrocytes, resveratrol increased the levels of reduced glutathione in cells and sulfhydryl groups in the membranes [18]. Unfortunately, the polyphenol possesses some disadvantages that hinder its therapeutic potential. It is characterized by poor aqueous solubility, low bioavailability, a fast metabolism, and low stability. The low stability of resveratrol is closely related to isomerization and photodegradation [19,20,21]. In order to overcome these drawbacks, many studies have been dedicated to the encapsulation of resveratrol in nanoparticles. For example, the chemical and physical stability of resveratrol was improved by its loading in lipid nanoparticles prepared from ethyl palmitate, polysorbate 60, and miglyol-812 [22]. Oral bioavailability and ocular permeability of resveratrol were increased by its embedding in casein and polycaprolactone nanoparticles [23,24]. Further, the incorporation of resveratrol into micelles based on poly(ε-caprolactone) and polyethyleneglycol enhanced its protective effects in a model of beta-amyloid peptide-induced damage in PC12 cells [25]. However, incorporation of the highly hydrophobic molecule of resveratrol into hydrophilic nanoparticles is a challenge, and there are very few resveratrol-loaded nanogel systems [26,27,28]. Furthermore, the use of natural polymers for their preparation is of particular interest due to their undeniable advantages.

The aim of this study was to encapsulate resveratrol in a nanogel prepared from natural compounds (citric acid and pentane-1,2,5-triol) and to characterize the obtained nanosystem. The ability of the nanogel to improve the protective effect of resveratrol against oxidative damage was evaluated in two types of cells, particularly neuroblastoma cell line and fibroblast cells. Furthermore, the protective effect of the encapsulated resveratrol was studied in a model of induced lipid peroxidation in rat liver and brain microsomes.

## 2. Results and Discussion

**Figure 1:**
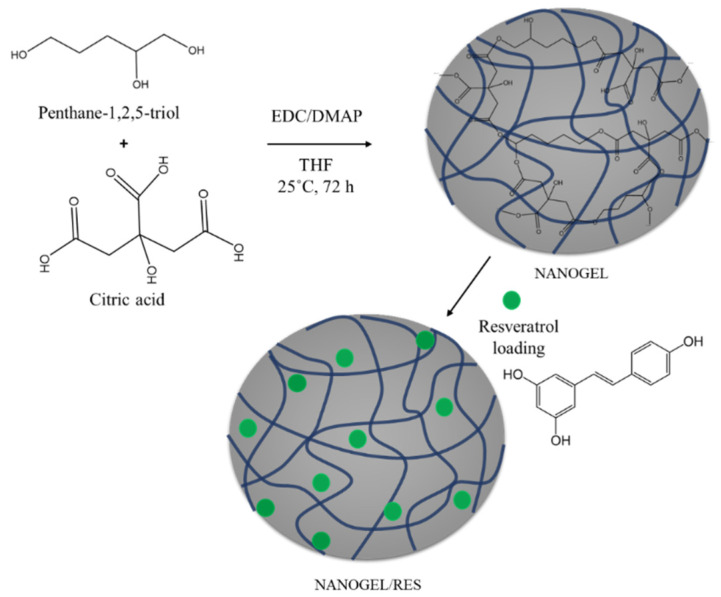
Preparation of resveratrol-loaded nanogels based on pentane-1,2,5-triol and citric acid.

The polymer nanogel was obtained via the precipitation polymerization reaction of two natural polyfunctional reagents—pentane-1,2,5-triol and citric acid. The ester bonds formed within the polymer network can be hydrolyzed under environmental conditions, making such materials biodegradable and suitable for biomedical applications [29]. The nanogel particles were synthesized at mild conditions via the Steglich esterification precipitation reaction in tetrahydrofuran (THF) at room temperature with the aid of N-ethyl-N′-(3-dimethylaminopropyl) carbodiimide as a coupling reagent and 4-(dimethylamino)-pyridine as a catalyst (Figure 1). Specifically, the reaction started in a solution, but after a certain time, the formed particles became insoluble in tetrahydrofuran and precipitated as a separate phase. The resulting nanogel was isolated, purified, and redispersed in deionized water. In the next step, resveratrol, dissolved in ethanol, was loaded into the nanogel via hydrophobic interactions between the drug molecules and hydrophobic segments of the polymer network (Figure 1).

**Figure 2:**
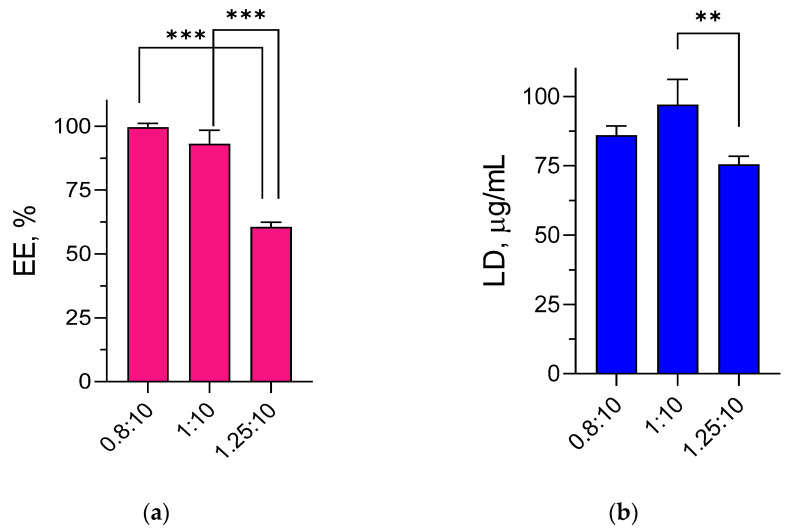
Encapsulation efficiency (EE) (**a**) and loading degree (LD) (**b**) of resveratrol-loaded nanogel prepared at different ratios between the drug and the polymers forming the nanogel; ** *p* < 0.01; *** *p* < 0.001 between different systems.

The loading of resveratrol in the nanogel dispersion was conducted in three different mass ratios between the active substance and the polymeric nanogel, namely 0.8:10, 1:10, and 1.25:10 (Figure 2a,b). As seen in Figure 2, a tendency for slightly higher encapsulation efficiency was observed in the nanogel prepared at a ratio of 0.8:10. However, a higher drug loading degree in the nanogel was obtained at a 1:10 ratio (adjusted *p* values of 0.0532 and 0.0652, respectively, between systems with ratios of 0.8:10 and 1:10). Significantly lower encapsulation efficiency and loading degree of resveratrol were observed when the ratio between resveratrol and polymeric nanogel was 1.25:10. It appeared that with further increase in the initial amount of resveratrol, its encapsulation into the nanogel decreased. The probable reason could be that the limit of nanogel groups for hydrophobic interactions has already been reached. Thus, the ratio 1:10 was considered optimal, and it was applied for the preparation of the nanogel for further experiments.

**Figure 3:**
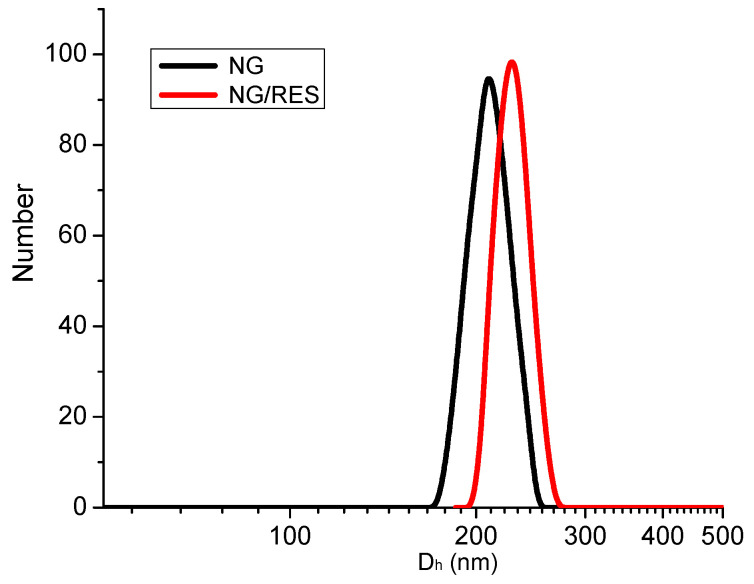
Size distribution plot of empty (NG) and resveratrol-loaded nanogel (NG/RES) particles (1:10).

The size distribution and zeta potential of empty and resveratrol-loaded nanogels were determined by dynamic light scattering (DLS) analysis at 25 °C (Figure 3 and Table 1). The empty nanogel was characterized by a monomodal size distribution and a hydrodynamic diameter of 207 ± 4 nm. The zeta potential value of nanogel particles was negative (Table 1) due to the presence of -COOH groups within the network. Loading of resveratrol slightly increased the nanogel size, polydispersity, and zeta potential (Table 1). Most probably, the inclusion of drug molecules within the carrier had a shielding effect, and the charge of the nanogel surface became neutral. However, the drug-loaded nanogel remained colloidally stable for more than one week since no precipitation was visible and the DLS plots (day 1 and day 7) were identical.

**Figure 4:**
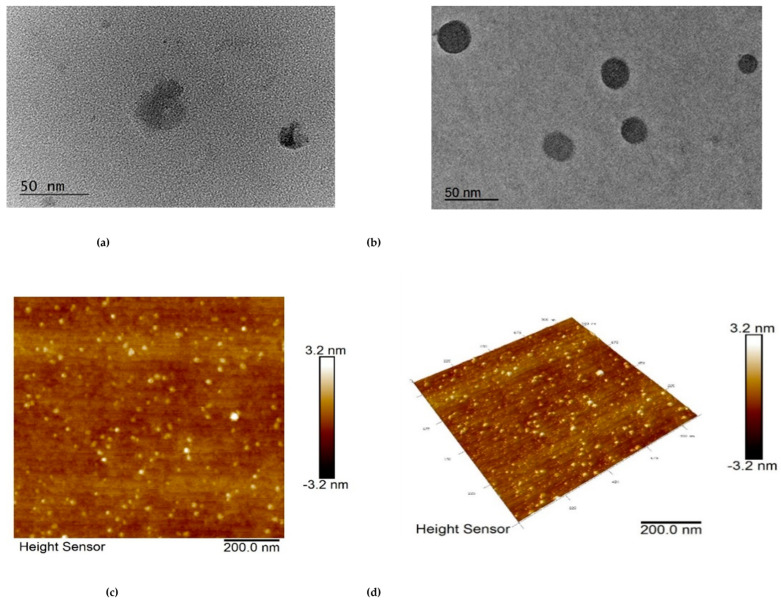
Transmission electron microscopy of empty (**a**) and resveratrol-loaded nanogel (**b**) and atomic force microscopy of 2D (**c**) and 3D (**d**) height images of resveratrol-loaded nanogel.

Transmission electron microscopy (TEM) was performed to evaluate the shape of the nanogel particles. The images revealed a spherical shape of the empty and drug-loaded nanogel particles (Figure 4a,b) and a smaller diameter than the one observed by DLS. Further, the surface morphology of the drug-loaded nanogel was investigated by atomic force microscopy (AFM). As can be seen from the image (Figure 4c,d), the dominant population of particles had a uniform spherical shape and nanoscopic dimensions. Similarly to TEM, the apparent diameter of the particles visualized by AFM was smaller than the one determined by DLS. In our opinion, a drying procedure during sample preparation led to dehydration and shrinkage of the particles, which was the reason for the observation of smaller sizes.

**Figure 5:**
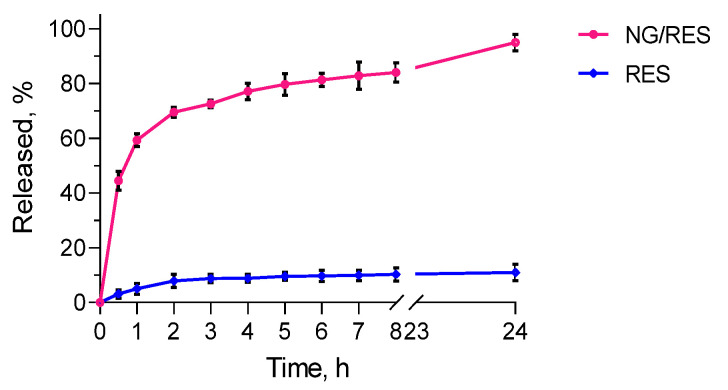
In vitro release profiles of pure (RES) and loaded into the nanogel resveratrol (NG/RES) in a phosphate buffer with pH = 7.4.

The in vitro release test of pure and encapsulated into the nanogel resveratrol was performed in a phosphate buffer with a pH of 7.4 (Figure 5). The drug release from the nanogel followed a biphasic pattern, with a burst effect at the beginning (70% for 2 h) and sustained release after that. To evaluate the mechanism of drug release, zero-order, first-order, and diffusion-controlled release models were used to analyze the data. The calculations showed that the correlation coefficient for first-order was higher (R^2^ = 0.9829) than that for zero-order (R^2^ = 0.704) and the Higuchi model (R^2^ = 0.9348). As seen in Figure 5, the release rate diminished after the initial burst, indicating that the rate depended on the remaining drug concentration. Further, it was found that the release of pure resveratrol was incomplete; in particular, not more than 11% was released for 24 h. In contrast, the entrapment of resveratrol into the nanogel provided its complete release. Thus, taking into consideration the poor water solubility of resveratrol (0.05 mg/mL) [20], it could be concluded that the nanogel system improved its biopharmaceutical behavior. This finding had practical importance related to the possible improvement of the bioavailability of resveratrol, which was reported as low [20]. 

**Figure 6:**
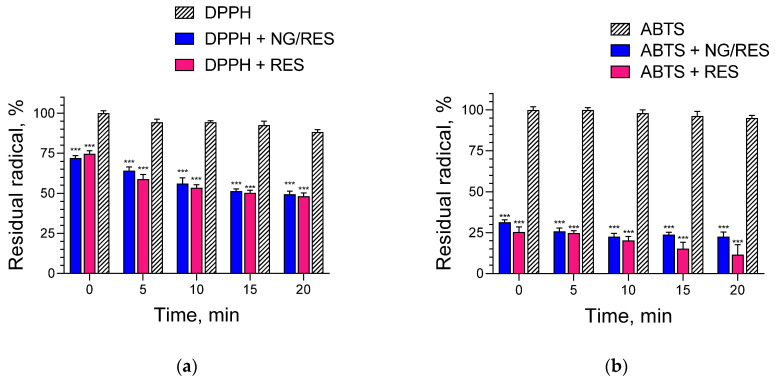
Scavenge activity of pure (RES) and encapsulated nanogel resveratrol (NG/RES) in a system containing DPPH (**a**) or ABTS (**b**) radicals. *** *p* < 0.001 vs. DPPH and ABTS groups.

Aiming to test the ability of pure and encapsulated resveratrol to scavenge free radicals, DPPH and ABTS assays were performed. The incubation of DPPH with pure and encapsulated resveratrol definitely decreased the concentration of DPPH radical in the solution (Figure 6a). The results showed that the radical scavenging activity of pure and encapsulated resveratrol was almost equal (no statistical significance between both groups). As shown, after 20 min of reaction, there was an approximately 50% reduction of the DPPH radical in both cases. Similarly, the results from the ABTS assay revealed the ability of pure and encapsulated resveratrol to scavenge the radical (Figure 6b). Statistical analyses showed that there was no significant difference between the two groups. Thus, it could be concluded that the encapsulation of resveratrol did not decrease its radical scavenging activity. These results were important, taking into consideration that in some cases, the incorporation of antioxidants into nanosystems could decrease their activity. For instance, the loading of kaempferol into two types of polymeric micelles with different cores and equal micellar coronas showed that the radical scavenging activity of the drug was different [30]. A significant decrease in activity was observed after kaempferol loading into micelles containing a poly(propylene oxide) core, whereas those containing a poly(ε-caprolactone) core preserved drug activity. Thus, the results indicated that the developed nanogel system in the present study was an appropriate carrier for resveratrol. It is important to note that the encapsulation could protect resveratrol from photolysis and consequently preserve its antioxidant activity during storage.

**Figure 7:**
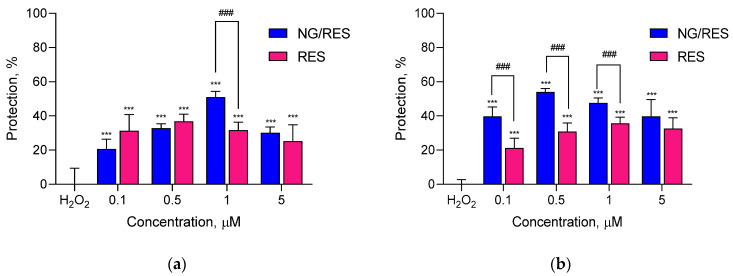
Protective effect of pure (RES) and encapsulated in nanogel resveratrol (NG/RES) against H_2_O_2_-induced oxidative stress in neuroblastoma SH-SY5Y (**a**) and fibroblast L929 cell line (**b**); *** *p* < 0.001 vs. H_2_O_2_-treated control; ### *p* < 0.001 between groups treated with pure and encapsulated resveratrol in corresponding concentrations.

Several disorders related to oxidative stress have been shown to be caused by the degeneration either of neurons due to their high susceptibility to free radical-related injuries or of fibroblasts due to their ubiquitous distribution and supportive roles in the organism [31,32]. In this view, the antioxidant activity of pure and encapsulated resveratrol was evaluated in a hydrogen peroxide (H_2_O_2_)-induced model of oxidative damage in two cell lines of different origin—human neuroblastoma SH-SY5Y cells and mouse L929 fibroblasts. The exposure of both cell lines to H_2_O_2_ resulted in morphological changes in the cells typical of apoptosis (data not shown). Regarding SH-SY5Y cells, treatment with pure and encapsulated resveratrol showed protection against H_2_O_2_-induced oxidative stress (Figure 7a). Interestingly, there was a statistically significant difference between the effects of pure and encapsulated drugs at 1 µM concentration (*p* < 0.001). This observation correlated with other studies that reported enhanced antioxidant effects of resveratrol encapsulated in different types of nanoparticles [33,34,35,36] as well as its neuroprotective effects in in vitro cell models [37].

Resveratrol may exert a beneficial effect in some genetic disorders with oxidative stress-related pathogenesis affecting the connective tissue and skin [38,39]. For this reason, the second type of cells selected were L929 fibroblasts. The incubation with pure and encapsulated resveratrol exerted protection to different degrees (Figure 7b). The encapsulated resveratrol (at 0.1, 0.5, and 1 µM) showed more pronounced and statistically significant protective effects on H_2_O_2_-damaged L929 cells compared to the pure drug (Figure 7b). This might be due to the capability of the nanosystem to improve the aqueous solubility of resveratrol, which further resulted in higher cellular uptake and enhanced activity. Our findings showed that the resveratrol-loaded nanogel could decrease oxidative stress in fibroblasts in this in vitro model system.

**Figure 8:**
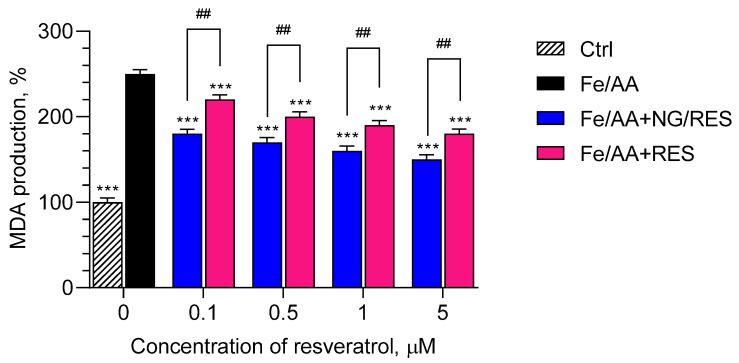
Effects of resveratrol-loaded nanogel (NG/RES) and pure resveratrol (RES) on the production of malondialdehyde (MDA) in conditions of iron/ascorbic acid (Fe/AA)-induced lipid peroxidation on rat liver microsomes. *** *p* < 0.001 vs. Fe/AA-treated group; ## *p* < 0.01 between the groups treated with encapsulated and pure resveratrol.

**Figure 9:**
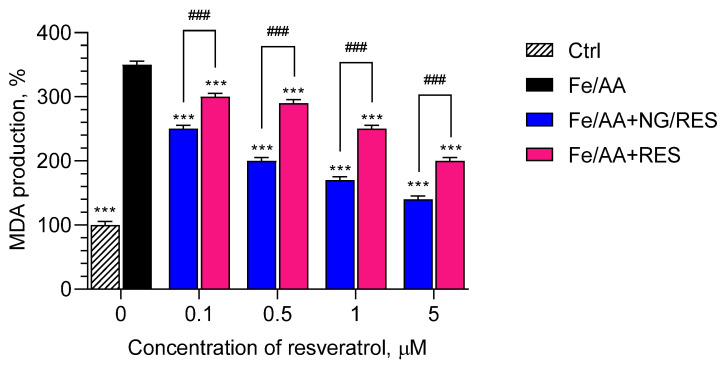
Effects of resveratrol-loaded nanogel (NG-RES) and pure resveratrol (RES) on the production of malondialdehyde (MDA) in conditions of iron/ascorbic acid (Fe/AA)-induced lipid peroxidation on rat brain microsomes. *** *p* < 0.001 vs. Fe/AA-treated group; ### *p* < 0.01 between the groups treated with encapsulated and pure resveratrol.

The protective effects of encapsulated and pure resveratrol were also studied in a model of non-enzyme iron/ascorbic acid (Fe/AA)-induced lipid peroxidation in isolated rat liver and brain microsomes. The first stage was to establish if the samples exerted a prooxidant effect on the microsomes. The treatment of both types of microsomes with empty nanogel, resveratrol-loaded nanogel, and pure resveratrol did not show prooxidant effects on non-treated microsomes (not shown). Further, the microsomes were treated with ferrous sulfate and ascorbic acid, aiming to provoke non-enzyme lipid peroxidation. The experimental conditions showed that the treatment increased malondialdehyde (MDA) production by 150% and 250% compared to the levels of non-treated liver and brain microsomes, respectively (Figure 8 and Figure 9). The pre-treatment with pure and encapsulated resveratrol revealed that it decreased the levels of malondialdehyde. The results on liver microsomes are presented in Figure 8. As seen, the treatment with the encapsulated resveratrol achieved statistically significant protection against lipid peroxidation compared to the treatment with the pure drug. The protection became stronger with the increase in concentration. In particular, at the highest concentration of encapsulated resveratrol (5 µM), the production of malondialdehyde decreased by 40% (corresponding to approx. 67% protection), whereas at the same concentration of pure resveratrol, the decrease of MDA was 28% (corresponding to approx. 47% protection).

Similar results were observed on the model of lipid peroxidation in brain microsomes (Figure 9). In this case, at a 5 µM concentration of resveratrol-loaded nanogel, the production of malondialdehyde decreased by 60% (corresponding to approx. 84% protection), whereas the treatment with pure resveratrol decreased MDA production by 43% (corresponding to approx. 60% protection). The comparison of both models showed that the protection in the brain microsomes by encapsulated resveratrol was 84%, whereas the protection in the liver microsomes was 67%. Thus, the protective effect of resveratrol-loaded nanogel was stronger in brain microsomes compared to liver microsomes.

## 3. Conclusions

Biodegradable nanogel particles, based on natural substances, were developed as a drug delivery system for the hydrophobic drug resveratrol. The nanogel particles provided high loading, improved solubility, and enhanced protective effects of encapsulated resveratrol against H_2_O_2_-induced oxidative stress, particularly in fibroblast cells. The protective effects of encapsulated and pure resveratrol against non-enzyme iron/ascorbic acid-induced lipid peroxidation in isolated rat liver and brain microsomes revealed significantly higher protection achieved with encapsulated resveratrol compared to the pure drug. Thus, the newly developed nanogel system was considered an appropriate platform for resveratrol application in oxidative stress-related disorders.

## 4. Materials and Methods

### 4.1. Materials

Trans-resveratrol was purchased from Sigma-Aldrich (FOT, Sofia, Bulgaria). Citric acid, 4-(dimethylamino)pyridine, and furfuryl alcohol were obtained from Sigma-Aldrich (FOT, Sofia, Bulgaria). Tetrahydrofuran (THF) (Sigma-Aldrich, FOT, Sofia, Bulgaria) was stirred overnight over calcium hydride and distilled prior to use. N-(3-dimethylaminopropyl)-Nʹ-ethylcarbodiimide hydrochloride (Merck, FOT, Sofia, Bulgaria) was used as received. Dulbecco’s Modified Eagle’s Medium, Roswell Park Memorial Institute 1640 Medium, fetal bovine serum (FBS), L-glutamine, 3-(4,5-dimethylthiazol-2-yl)-2,5-diphenyltetrazolium bromide (MTT), 2,2-diphenyl-1-picrylhydrazyl (DPPH), and 2,2′-azino-bis(3-ethylbenzothiazoline-6-sulfonic acid) (ABTS) were purchased from Sigma-Aldrich (Merck KGaA, Darmstadt, Germany). Dialysis membrane (standard-grade regenerated cellulose, 10,000 MWCO, Spectrum Labs) was obtained from Fisher Scientific (Göteborg, Sweden). The neuroblastoma cell line SH-SY5Y and murine L929 cell line were acquired from the European Collection of Cell Cultures (ECACC, Salisbury, UK).

### 4.2. Preparation and Drug Loading of Nanogel

One of the monomers, pentane-1,2,5-triol, was synthesized according to a procedure reported in a previous paper [40]. The nanogel was prepared as described elsewhere [29]. In brief, the nanogel particles were obtained via the esterification precipitation reaction of pentane-1,2,5-triol and citric acid in anhydrous tetrahydrofuran, using 1-ethyl-3-(3-dimethylaminopropyl) carbodiimide hydrochloride as a coupling reagent and 4-(dimethylamino)-pyridine as a catalyst. The reaction was carried out for 72 h in an inert atmosphere at room temperature. The resulting nanogel was purified by filtration and subsequent dialysis (MWCO = 3500) against deionized water for 5 days. The final product was collected by freeze-drying.

The loading of resveratrol in the obtained nanogel was conducted via solvent evaporation. Briefly, a solution of resveratrol in ethanol in different concentrations was added to the aqueous dispersion of the nanogel. The system was stirred at 700 rpm for 2 h in order to evaporate the ethanol. Then the loaded nanogel dispersion was filtered (0.45 µM) and the non-encapsulated resveratrol in the rinsing filter fractions was determined spectrophotometrically at λ = 306 nm (Thermo Fisher Scientific, Waltham, MA, USA). The concentration of resveratrol was calculated using a standard curve obtained in the range of 2–10 µg/mL (r > 0.9996). The encapsulation efficiency (EE) and loading degree (LD) of resveratrol were determined using the following equations:

EE = (Total mass of drug − non-encapsulated drug)/Total mass of drug(1)

LD = (Total mass of drug − non-encapsulated drug)/Volume of drug loaded nanogel dispersion(2)

### 4.3. Physicochemical Characterization of the Nanogel

The hydrodynamic diameter and dispersity index of empty and drug-loaded nanogels were determined using the Zetasizer NanoBrook 90Plus PALS instrument, equipped with a 35 mW red diode laser (λ = 640 nm) at a scattering angle of 90°. The zeta potential was determined by electrophoretic light scattering (PALS method) at a scattering angle of 15°. The measurements were performed in triplicate at 25 °C at a sample concentration of 1.0 g/L. Transmission electron microscopy (TEM) was conducted using a HR STEM JEOL JEM 2100 (Tokyo, Japan). The morphology of resveratrol-loaded nanogel was studied via atomic force microscopy (AFM) analyses with a Bruker Dimension Icon microscope under ambient conditions at a 1.00 Hz scan rate. An amount of 2 μL of solution (1 g/L) was placed onto a freshly cleaned glass substrate and spin-coated at 2000 rpm for a minute. The measurements were performed in ScanAsyst mode.

### 4.4. In Vitro Release Tests of Encapsulated and Pure Resveratrol

The in vitro release tests of the encapsulated and pure resveratrol were conducted via dialysis in a phosphate buffer (pH = 7.4) containing 10% ethanol (*n* = 3). Briefly, 1.5 mL of the nanogel dispersion, containing 0.316 mg resveratrol, or an aqueous suspension containing the same concentration, were introduced into a dialysis membrane. The membrane was placed in 20 mL of the medium under gentle shaking at 37 °C (IKA Labortechnik HS-B20, Staufen, Germany). At predetermined time intervals, samples of 2 mL were taken from the acceptor phase, and the concentration of resveratrol was determined UV-spectrophotometrically at 306 nm (Thermo Fisher Scientific, Waltham, MA, USA). The equivalent amount of fresh medium was added back in order to maintain sink conditions.

The mechanism of drug release was evaluated via fitting the data to zero-order (Equation (3)), first-order (Equation (4)), and Higuchi models (Equation (5)):

C_t_ = C_0_ + K_0_t (3)

where C_t_ represents the amount of active agent released during the time t; C_0_ is the initial concentration of the drug released; and K_0_ is the zero-order rate constant.

ln(C_i_ − C_t_) = ln(C_i_) − K_1_t (4)

where C_t_ represents the amount of active agent released during the time t; C_i_ is the initial concentration of the drug before release; and K_1_ is the first-order rate constant.

C_t_ = Kt^1/2^ (5)

where C_t_ is the amount of drug released during the time t and K is the release constant of Higuchi.

### 4.5. DPPH and ABTS Assay

The DPPH assay was conducted after optimizing the previously described protocol [41]. Briefly, 100 µL of resveratrol-loaded nanogel dispersion (78 µg/mL) and a hydroalcoholic solution of pure resveratrol in the same concentration were added to a 100 µL ethanol solution of DPPH (440 µg/mL). Immediately after that, the absorbance of the reduced form of DPPH was measured in a multiplate reader, Synergy 2 (BioTek Instruments, Inc., Highland Park, Winooski, VT, USA), at 517 nm and 25 °C.

The ABTS assay was performed after modification of previously described procedures [42,43]. A stock solution of ABTS was mixed with potassium persulfate in water, and the mixture was incubated in a dark place at 25 °C for 16 h. Therefore, 100 µL of resveratrol-loaded nanogel dispersion (78 µg/mL) or a hydroalcoholic solution of pure resveratrol in the same concentration were added to 100 µL of a 5% alcoholic solution of ABTS. The absorbance of the samples was measured in a multiplate reader, Synergy 2 (BioTek Instruments, Inc., Highland Park, Winooski, VT, USA), at 734 nm and 30 °C.

### 4.6. Protective Effects on In Vitro Cell Models

The protective effects of nanogel and pure resveratrol (in DMSO) were evaluated in a hydrogen peroxide (H_2_O_2_)-induced model of oxidative stress on two cell lines from different origins—human neuroblastoma SH-SY5Y cells and murine L929 fibroblasts. The cells were seeded in 96-well plates at a cell density of 3.5 × 10^4^ for SH-SY5Y and 2 × 10^4^ for L929 and incubated overnight at standard conditions of 37 °C, 5% CO_2_, and high humidity (Esco CelCulture^®^ CO₂ Incubator, CCL-170B-8-IVF, Esco Micro Pte. Ltd., Singapore). After 24 h of incubation, the SH-SY5Y cells were treated for 90 min and the L929 cells for 2 h with resveratrol-loaded nanogel and pure resveratrol (0.1, 0.5, 1, and 5 µM). Thereafter, 1 mM H_2_O_2_ (10 min) and 500 µM H_2_O_2_ (30 min) were applied to SH-SY5Y and L929 cells, respectively. Each well was then washed with PBS containing Ca^2+^ and Mg^2+^ and fresh medium was added. After 24 h of incubation, a solution of MTT (10 mg/mL in PBS) was added to each well, and the plates were incubated at 37 °C for 3 h. After that, the MTT solution was carefully aspirated, and the formed formazan crystals were dissolved by the addition of 100 μL of DMSO. The absorbance was measured in a multiplate reader, Synergy 2 (BioTek Instruments, Inc., Highland Park, Winooski, VT, USA), at 570 nm (690 nm for background absorbance).

### 4.7. Animals and Isolation of Rat Liver and Brain Microsomes

Male Wistar rats (body weight 200–220 g) were purchased from the National Breeding Centre, Sofia, Bulgaria. The animals were housed under standard laboratory conditions (20 ± 2 °C and humidity 72 ± 4%) with free access to water and standard pelleted rat food (ISO 9001:2008). All procedures performed were approved by the Bulgarian Food Safety Agency with Permission N° 273/ valid till 2025.

For the isolation of liver microsomes, the animals (fasted overnight) were sacrificed by cervical decapitation, and the livers were perfused with 1.15% KCl and homogenized with four volumes of ice-cold potassium phosphate buffer (0.1 M, pH = 7.4). The liver homogenate was centrifuged at 9000× *g* for 30 min at 4 °C, and the resulting post-mitochondrial fraction was centrifuged again at 105,000× *g* for 60 min at 4 °C. The microsomal pellets were resuspended in 0.1 M potassium phosphate buffer (pH = 7.4), containing 20% glycerol. Aliquots of liver microsomes were stored at −70 °C until use [44].

For the isolation of rat brain microsomes, the brain was homogenized in 0.1 M Tris buffer containing 0.1 mM dithiothreitol, 0.1 mM phenylmethylsulfonyl fluoride, 0.2 mM EDTA, 1.15% KCl, and 20% (*v*/*v*) glycerol. The homogenate was centrifuged twice at 17,000× *g* for 30 min. The supernatants from both centrifugations were combined and centrifuged twice at 100,000× *g* for 1 h. The resulting pellet was frozen in the Tris buffer and stored until use [45].

The content of microsomal protein was determined according to the method of Lowry, using bovine serum albumin as a standard [46].

### 4.8. Iron/Ascorbic Acid Induced Lipid Peroxidation In Vitro

The isolated liver/brain microsomes (1 mg/mL) were preincubated with encapsulated and pure resveratrol (0.1–5 μM) for 30 min at 37 °C. Then, the lipid peroxidation started with the incubation of microsomes with 20 μM FeSO_4_ and 500 μM ascorbic acid for 30 min [47]. The lipid peroxidation was stopped by adding a mixture of 1 mL of 25% (*w*/*v*) trichloroacetic acid (TCA) and 1 mL of 0.67% 2-thiobarbituric acid (TBA) to the microsomes (100 °C, 20 min), and after that, the absorbance was measured at 535 nm (Spectro UV-VIS Split spectrophotometer). The amount of malondialdehyde (MDA) was calculated using a molar extinction coefficient of 1.5 × 10^5^ M^−1^cm^−1^ [47]. In order to determine the protective effects of encapsulated and pure resveratrol, the values were normalized, considering the negative control (non-treated microsomes) as 100% protection and the positive control (Fe/AA-treated) as 0% protection.

### 4.9. Statistical Analysis

All experiments were performed in triplicate. The results were expressed as mean values ± SD. GraphPad Prism 8 software (Dotmatics, San Diego, CA, USA) was used for the statistical analyses. A one-way ANOVA with Tukey’s multiple comparisons post-test was applied in order to compare the values of encapsulation efficiency and loading degree in the systems prepared at different ratios. A one-way ANOVA with Dunnett’s multiple comparison post-test was conducted, aiming to make comparisons between the treatments and DPPH-, ABTS-, H_2_O_2_- and Fe/AA-treated controls. Furthermore, multiple *t*-tests with the Holm–Sidak correction were applied for comparison between the groups in all experiments.

## Figures and Tables

**Table 1:** Data from dynamic light scattering and PALS measurements of empty (NG) and resveratrol-loaded nanogels (NG/RES).

Sample Code	D_h_ (nm) *	ζ-Potential (mV) ***	DI *
NG	207 ± 4	−9.2 ± 1.2	0.33 ± 0.017
NG/RES	220 ± 4	2.9 ± 1.0	0.39 ± 0.020

* *p* < 0.05; *** *p* < 0.001 between different systems.

## Data Availability

Not applicable.
